# Supplementary material for: Health Needs and Their Relationship with Life Expectancy in People with and without Intellectual Disabilities in England
Source: Int J Environ Res Public Health. 2022 May 28;19(11):6602. doi: 10.3390/ijerph19116602 (PMC9180100; doi:10.3390/ijerph19116602)
Supplement: Supplementary file 1 [file ijerph-19-06602-s001.zip › ijerph-1703878-supplementary.pdf]

Health needs and their relationship with life expectancy in people with and without intellectual disabilities in England

Supplementary material

Table S1: RECORD[a] checklist (Reporting of studies conducted using observational routinely-collected data; https://www.record-statement.org/checklist.php)

	Item No.	STROBE items	Location in original manuscript where items are reported		RECORD items	Location in manuscript where items are reported	
**Title and Abstract**							
	1	(a) Indicate the study's design with a commonly used term in the title or the abstract	☑	p.2	RECORD 1.1: The type of data used should be specified in the title or abstract. When possible, the name of the databases used should be included.	☑ Source of data (CPRD) cited in **Abstract**	p.2
		(b) Provide in the abstract an informative and balanced summary of what was done and what was found	☑	p.2	RECORD 1.2: If applicable, the geographic region and timeframe within which the study took place should be reported in the title or abstract.	☑ Calendar periods (2017–2019) listed in **Abstract**	p.2
					RECORD 1.3: If linkage between databases was conducted for the study, this should be clearly stated in the title or abstract	☑ Linked hospital/mortality data mentioned in the **Abstract**	p.2
**Introduction**							
Background rationale	2	Explain the scientific background and rationale for the investigation being reported	☑ **Introduction**	p.3			
Objectives	3	State specific objectives, including any prespecified hypotheses	☑ **Introduction**	p.3			
**Methods**							
Study Design	4	Present key elements of study design early in the paper	☑ See **Data sources**	p.3			

Setting	5	Describe the setting, locations, and relevant dates, including periods of recruitment, exposure, follow-up, and data collection	☑ See **Data sources** and **Statistical analyses**	p.4&7			
**Participants**	6	(a) *Cohort study* - Give the eligibility criteria, and the sources and methods of selection of participants. Describe methods of follow-up<br>*Case-control study* - Give the eligibility criteria, and the sources and methods of case ascertainment and control selection. Give the rationale for the choice of cases and controls<br>*Cross-sectional study* - Give the eligibility criteria, and the sources and methods of selection of participants	☑ Inclusion criteria listed in **Sources of Data** Additional cohort entry and exit criteria are listed under **Statistical Methods**	p. 4&7	RECORD 6.1: The methods of study population selection (such as codes or algorithms used to identify subjects) should be listed in detail. If this is not possible, an explanation should be provided.	☑ All codes are listed in the supplementary material	(S2) p.11
		(b) *Cohort study* For matched studies, give matching criteria and number of exposed and unexposed<br>*Case-control study* - For matched studies, give matching criteria and the number of controls per case	N/A		RECORD 6.2: Any validation studies of the codes or algorithms used to select the population should be referenced. If validation was conducted for this study and not published elsewhere, detailed methods and results should be provided.	☑ Codes were identified from the literature, free-text searching of Read code descriptions and clinical opinion, as described.	Table S2 p.10–35
					RECORD 6.3: If the study involved linkage of databases, consider use of a flow diagram or other graphical display to demonstrate the data linkage	☑ Figure S1; p.36	-

					process, including the number of individuals with linked data at each stage.		
Variables	7	Clearly define all outcomes, exposures, predictors, potential confounders, and effect modifiers. Give diagnostic criteria, if applicable.	☑ See **Definition of intellectual disabilities and health needs**	p.4	RECORD 7.1: A complete list of codes and algorithms used to classify exposures, outcomes, confounders, and effect modifiers should be provided. If these cannot be reported, an explanation should be provided.	☑ See supplementary material.	(S2) p.11
Data sources/ measurement	8	For each variable of interest, give sources of data and details of methods of assessment (measurement). Describe comparability of assessment methods if there is more than one group	☑ See **Definition of intellectual disabilities and health needs**	p.4			
Bias	9	Describe any efforts to address potential sources of bias	☑ Errors in prevalence estimates mitigated by excluding most recent diagnoses >10years from cohort entry. Immortal time bias reduced by including treating intellectual disability as an age(time)-dependent covariate	p.4 & 5			
Study size	10	Explain how the study size was arrived at	☒ All of the exposed population on the CPRD meeting eligibility criteria were selected. Feasibility counts for	-			

			sample size are included in the cited ISAC protocol number.		
Quantitative variables	11	Explain how quantitative variables were handled in the analyses. If applicable, describe which groupings were chosen, and why	☑ See **Data Sources**	p.3	
Statistical methods	12	(a) Describe all statistical methods, including those used to control for confounding	☑ See **Statistical methods**	p.5	
		(b) Describe any methods used to examine subgroups and interactions	☑ Subjects were stratified by health need. Interaction term fitted – described in **Statistical methods**	p.5	
		(c) Explain how missing data were addressed	☒ N/A for this study	-	
		(d) *Cohort study* - If applicable, explain how loss to follow-up was addressed *Case-control study* - If applicable, explain how matching of cases and controls was addressed *Cross-sectional study* - If applicable, describe analytical methods taking account of sampling strategy	☑ Right censoring strategy described in **Statistical methods**	p.5	
		(e) Describe any sensitivity analyses	☒ N/A for this study.	-	

Data access and cleaning methods					RECORD 12.1: Authors should describe the extent to which the investigators had access to the database population used to create the study population.	☑ This is an established research database that has been quality assessed and checked for internal validity. Relevant papers have been cited in **Data sources**.	p.3
					RECORD 12.2: Authors should provide information on the data cleaning methods used in the study.	☑ Relevant codes have been described for this study and how they were used. Data cleaning was minimal because measurements/ results were not used for this analysis.	Table S2 p.10–35
Linkage					RECORD 12.3: State whether the study included person-level, institutional-level, or other data linkage across two or more databases. The methods of linkage and methods of linkage quality evaluation should be provided.	☑ Person-level linkage was used for this analysis as specified in the methods (**Data Sources**)	p.3
**Results**							
Participants	13	(a) Report the numbers of individuals at each stage of the study (*e.g.*, numbers potentially eligible, examined for eligibility, confirmed eligible, included in the study, completing follow-up, and analysed)	☑ See Table 1. Also see flowchart (supplementary material Figure S1)	Figure S1; p.36	RECORD 13.1: Describe in detail the selection of the persons included in the study (*i.e.,* study population selection) including filtering based on data quality, data availability and linkage. The selection of included persons can be described in the text and/or by means of the study flow diagram.	☑ A description of subjects is in Table 1 – and by individual health need is in the supplementary tables (Table S3). The flow diagram is also shown in supplementary figure S1.	Table S3; 37–40 Figure S1; p.36

		(b) Give reasons for non-participation at each stage.<br><br>(c) Consider use of a flow diagram	☑ See flowchart<br><br>☑ See flowchart	Figure S1; p.36<br><br>-			
Descriptive data	14	(a) Give characteristics of study participants (*e.g.*, demographic, clinical, social) and information on exposures and potential confounders<br>(b) Indicate the number of participants with missing data for each variable of interest<br><br>(c) *Cohort study* - summarise follow-up time (*e.g.*, average and total amount)	☑ Baseline characteristics of the study population are shown in Table1<br><br>N/A<br><br>☑ Table 1	p.5<br><br>-<br><br>p.5			
Outcome data	15	*Cohort study* - Report numbers of outcome events or summary measures over time<br>*Case-control study* - Report numbers in each exposure category, or summary measures of exposure<br>*Cross-sectional study* - Report numbers of outcome events or summary measures	☑ Table 1	p.5			
	16	(a) Give unadjusted estimates and, if applicable, confounder-adjusted estimates and their precision (e.g., 95% confidence interval). Make clear which confounders were adjusted for and why they were included	☑ 95% confidence intervals reported in the figures and Table 2.	p.9 & 10			

		(b) Report category boundaries when continuous variables were categorized<br>(c) If relevant, consider translating estimates of relative risk into absolute risk for a meaningful time period	N/A (exact age)<br>☑ Life years lost and additional years expected to live presented – often conceptually easier to understand				
Other analyses	17	Report other analyses done—e.g., analyses of subgroups and interactions, and sensitivity analyses	N/A	-			
**Discussion**							
Key results	18	Summarise key results with reference to study objectives	☑ See **Discussion**	p.11			
Limitations	19	Discuss limitations of the study, taking into account sources of potential bias or imprecision. Discuss both direction and magnitude of any potential bias	☑ See **Discussion**	p.11–12	RECORD 19.1: Discuss the implications of using data that were not created or collected to answer the specific research question(s). Include discussion of misclassification bias, unmeasured confounding, missing data, and changing eligibility over time, as they pertain to the study being reported.	☑ See strengths and limitations	p.11–12
Interpretation	20	Give a cautious overall interpretation of results considering objectives, limitations, multiplicity of analyses, results from similar studies, and other relevant evidence	☑ See **Discussion**	p.11–13			

Generalisability	21	Discuss the generalisability (external validity) of the study results	☑ See **Discussion**	p.11–13			
**Other Information**							
Funding	22	Give the source of funding and the role of the funders for the present study and, if applicable, for the original study on which the present article is based	☑ See **Funding**	p.14			
Accessibility of protocol, raw data, and programming code					RECORD 22.1: Authors should provide information on how to access any supplemental information such as the study protocol, raw data, or programming code.	☑ The CPRD's ISAC study protocol ID is listed and available to view on-line (**Data sources**). Raw data are not available and an explanation is given for this in the **Data availability** statement.	p.14–15

Checklist is protected under Creative Commons Attribution (CC BY) licence.

[a] Reference: Benchimol EI, Smeeth L, Guttmann A, Harron K, Moher D, Petersen I, Sørensen HT, von Elm E, Langan SM, the RECORD Working Committee. The REporting of studies Conducted using Observational Routinely-collected health Data (RECORD) Statement. *PLoS Medicine* 2015; 2(10): e1001885. https://doi.org/10.1371/journal.pmed.1001885

Table S2: Diagnostic and classification codes for intellectual disabilities, ethnicity and severe health needs investigated

**Primary care Read codes for intellectual disability diagnoses** (identified from Tests, Referrals or Clinical files)	
**Read Code**	**Read Code Description**
C31yX00	Disorder of glycoprotein metabolism, unspecified
C372000	Hypoxanthine-guanine-phosphoribosyltransferase deficiency
C372011	Lesch - Nyhan syndrome
C372300	Lesch-Nyhan syndrome
C372z00	Other disorder of purine or pyrimidine metabolism NOS
E141000	Active disintegrative psychoses
E141100	Residual disintegrative psychoses
E141z00	Disintegrative psychosis NOS
Eu70000	[X]Mld mental retard with statement no or min impairm behav
Eu70100	[X]Mld mental retard sig impairment behav req attent/treatmt
Eu70y00	[X]Mild mental retardation, other impairments of behaviour
Eu70z00	[X]Mild mental retardation without mention impairment behav
Eu71000	[X]Mod mental retard with statement no or min impairm behav
Eu71100	[X]Mod mental retard sig impairment behav req attent/treatmt
Eu71y00	[X]Mod retard oth behav impair
Eu71z00	[X]Mod mental retardation without mention impairment behav
Eu72000	[X]Sev mental retard with statement no or min impairm behav
Eu72100	[X]Sev mental retard sig impairment behav req attent/treatmt
Eu72y00	[X]Severe mental retardation, other impairments of behaviour
Eu72z00	[X]Sev mental retardation without mention impairment behav
Eu73000	[X]Profound ment retrd wth statement no or min impairm behav
Eu73100	[X]Profound ment retard sig impairmnt behav req attent/treat
Eu73y00	[X]Profound mental retardation, other impairments of behavr
Eu73z00	[X]Prfnd mental retardation without mention impairment behav
Eu7y000	[X]Oth mental retard with statement no or min impairm behav
Eu7y100	[X]Oth mental retard sig impairment behav req attent/treatmt
Eu7yy00	[X]Other mental retardation, other impairments of behaviour
Eu7yz00	[X]Other mental retardation without mention impairment behav
Eu7z000	[X]Unsp mental retard with statement no or min impairm behav
Eu7z100	[X]Unsp mentl retard sig impairment behav req attent/treatmt
Eu7zy00	[X]Unspecified mental retardatn, other impairments of behav
Eu7zz00	[X]Unsp mental retardation without mention impairment behav
Eu81400	[X]Moderate learning disability
Eu81500	[X]Severe learning disability
Eu81600	[X]Mild learning disability
Eu81700	[X]Profound learning disability
Eu81800	[X]Specific learning disability
Eu81z00	[X]Developmental disorder of scholastic skills, unspecified
Eu81z11	[X]Learning disability NOS
Eu81z12	[X]Learning disorder NOS
Eu81z13	[X]Learn acquisition disab NOS

Eu84112	[X]Mental retardation with autistic features
Eu84200	[X]Rett's syndrome
Eu84300	[X]Other childhood disintegrative disorder
Eu84311	[X]Dementia infantalis
Eu84312	[X]Disintegrative psychosis
Eu84313	[X]Heller's syndrome
Eu84400	[X]Overactive disorder assoc mental retard/stereotype movts
P22yz00	Other reduction deformity of brain NOS
PJ33100	Deletion of long arm of chromosome 18
PJ33111	18p- syndrome
PJ33200	Deletion of short arm of chromosome 18
PJ33211	18q- syndrome
PJ33300	Smith-Magenis syndrome
PJ33400	Jacobsen syndrome
PJ33500	Greig cephalopolysyndactyly syndrome
PJ33700	3p deletion syndrome
PJ33800	Chromosome 4q deletion syndrome
PJ33900	Langer-Giedion syndrome
PJ33A00	Kleefstra syndrome
PJ50000	Trisomy 6
PJ50100	Trisomy 7
PJ50200	Trisomy 8
PJ50300	Trisomy 9
PJ50400	Trisomy 10
PJ50500	Trisomy 11
PJ50600	Trisomy 12
PJ50700	Other trisomy C syndromes
PJ50800	Trisomy 22
PJ50w00	Whole chromosome trisomy, meitotic nondisjunction
PJ50x00	Whole chromosome trisomy, mosaicism
PJ50x11	Whole chromosome trisomy, mitotic nondisjunction
PJ50y00	Other specified whole chromosome trisomy syndrome
PJ50z00	Whole chromosome trisomy syndrome NOS
PJ51000	Major partial trisomy
PJ51100	Minor partial trisomy
PJ51200	10q partial trisomy syndrome
PJ51300	Trisomy 4p syndrome
PJ51400	Trisomy 9p syndrome
PJ51500	15q partial trisomy syndrome
PJ51z00	Partial trisomy syndrome NOS
PJ52300	Triploidy
PJ52400	Polyploidy
PJ52z00	Trisomy of autosomes NEC NOS
PJyy200	Fragile X chromosome
PJyy400	Fragile X syndrome
PKy6100	Cockayne syndrome

PKy9300	Prader - Willi syndrome
PKyz.11	Cockayne's syndrome
PKyz511	Angelman syndrome
PKyz700	Angelman's syndrome
Pyu0200	[X]Other reduction deformities of brain
Pyu0300	[X]Other specified congenital malformations of brain
PyuA000	[X]Oth specif trisomies & partial trisomies of autosomes
R034y11	[D]Global retardation
ZL1B500	Under care of psychiatrist for mental handicap
918e.00	On learning disability register
C031.00	Goitrous cretin
C0A..00	Congenital iodine deficiency syndrome
C0A0.00	Congenital iodine-deficiency syndrome, neurological type
C0A1.00	Congenital iodine-deficiency syndrome, myxoedematous type
C301.00	Phenylketonuria
C372.00	Other disorders of purine and pyrimidine metabolism
C377.00	Disorders of glycoprotein metabolism
E141.00	Disintegrative psychosis
E3..00	Mental retardation
E30..00	Mild mental retardation, IQ in range 50-70
E31..00	Other specified mental retardation
E310.00	Moderate mental retardation, IQ in range 35-49
E311.00	Severe mental retardation, IQ in range 20-34
E312.00	Profound mental retardation with IQ less than 20
E31z.00	Other specified mental retardation NOS
E3y..00	Other specified mental retardation
E3z..00	Mental retardation NOS
Eu7..00	[X]Mental retardation
Eu70.00	[X]Mild mental retardation
Eu71.00	[X]Moderate mental retardation
Eu72.00	[X]Severe mental retardation
Eu73.00	[X]Profound mental retardation
Eu7y.00	[X]Other mental retardation
Eu7z.00	[X]Unspecified mental retardation
P01..00	Craniorachischisis
P02..00	Iniencephaly
P22..00	Reduction deformities of brain
P224.00	Arhinencephaly
P225.00	Holoprosencephaly
P22y.00	Other specified reduction deformities of brain
P22z.00	Reduction deformities of brain NOS
PJ0..00	Down's syndrome - trisomy 21
PJ00.00	Trisomy 21, meiotic nondisjunction
PJ02.00	Trisomy 21, translocation
PJ0z.00	Down's syndrome NOS
PJ1..00	Patau's syndrome - trisomy 13

PJ10.00	Trisomy 13, meiotic nondisjunction
PJ11.00	Trisomy 13, mosaicism
PJ12.00	Trisomy 13, translocation
PJ1z.00	Patau's syndrome NOS
PJ2..00	Edward's syndrome - trisomy 18
PJ20.00	Trisomy 18, meiotic nondisjunction
PJ21.00	Trisomy 18, mosaicism
PJ22.00	Trisomy 18, translocation
PJ2z.00	Edward's syndrome NOS
PJ30.00	Antimongolism syndrome
PJ31.00	Cri-du-chat syndrome
PJ32.00	Deletion of short arm of chromosome 4
PJ3z.00	Monosomies and deletions from the autosomes NOS
PJ50.00	Whole chromosome trisomy syndromes
PJ51.00	Partial trisomy syndromes
PJ52.00	Trisomies of autosomes NEC
PJ9..00	Mowat-Wilson syndrome
PK5..00	Tuberous sclerosis
PKy4.00	William syndrome
ZS34.00	Developmental disorder of scholastic skill
E3...00	Mental retardation
C03..11	Cretinism
C372.11	Lesch - Nyhan syndrome
E141.11	Heller's syndrome
E30..11	Educationally subnormal
E310.11	Imbecile
E312.11	Idiocy
Eu70.11	[X]Feeble-mindedness
Eu71.11	[X]Moderate mental subnormality
Eu72.11	[X]Severe mental subnormality
Eu73.11	[X]Profound mental subnormality
Eu7z.11	[X]Mental deficiency NOS
PJ0..11	Mongolism
PJ01.11	Trisomy 21, mitotic nondisjunction
PJ02.11	Partial trisomy 21 in Down's syndrome
PJ0z.11	Trisomy 21 NOS
PJ11.11	Trisomy 13, mitotic nondisjunction
PJ12.11	Partial trisomy 13 in Patau's syndrome
PJ1z.11	Trisomy 13 NOS
PJ21.11	Trisomy 18, mitotic nondisjunction
PJ22.11	Partial trisomy 18 in Edward's syndrome
PJ2z.11	TRISOMY 18 NOS
PJ30.11	Deletion of long arm of chromosome 21
PJ31.11	Deletion of short arm of chromosome 5
PJ32.11	Wolff - Hirschorn syndrome
PKy0.11	Prader-Willi Syndrome

ZS34.11	Learning disability
C03z.12	Cretinism
E30..12	Feeble-minded
Eu70.12	[X]Mild mental subnormality
Eu7z.12	[X]Mental subnormality NOS
PJ0..12	Trisomy 21
PKy0.12	Prader-Willi syndrome
E30..13	Moron
PJ0..13	Trisomy 22
8Ce6.00	Preferred place of care - learning disability unit
9HB..00	Learning disabilities administration status
9HB0.00	Learning disabilities health action plan declined
9HB1.00	Learning disabilities health action plan offered
9HB2.00	Learning disabilities health action plan reviewed
9HB3.00	Learning disabilities health assessment
9HB4.00	Learning disabilities health action plan completed
9HB5.00	Learning disabilities annual health assessment
9HB6.00	Learning disabilities annual health assessment declined
9HB7.00	Did not attend learning disabilities annual health assessmnt
9hL..00	Exception reporting: learning disability quality indicators
9hL0.00	Exc learn disability quality indicators: informed dissent
9hL1.00	Exc learn disability quality indicators: patient unsuitable
9mA..00	Learning disability annual health check invitation
9mA0.00	Learning disability annual health check verbal invitation
9mA1.00	Learning disability annual health check telephone invitation
9mA2.00	Learning disability annual health check letter invitation
9mA2000	Learning disability annual health check invtation 1st letter
9mA2100	Learning disability annual health check invtation 2nd letter
9mA2200	Learning disability annual health check invtation 3rd letter
9Nh4.00	Under care of community learning disability team
9HB6.11	Learning disabilities annual health check declined
9HB7.11	Did not attend learning disabilities annual health check
13Z3.00	Low I.Q.
69DB.00	Learning disability health examination
94Z9.00	Preferred place of death: learning disability unit
6664	Mental handicap problem

**Primary care codes for ethnicity status**		
**Read Code**	**Read code description**	**Ethnic group**
916E.00	Patient ethnicity unknown	Ethnic group not specified
9i...00	Ethnic category - 2001 census	Ethnic group not specified
9i0..00	British or mixed British - ethnic category 2001 census	Ethnic group not specified
9i00.00	White British - ethnic category 2001 census	White
9i1..00	Irish - ethnic category 2001 census	White
9i10.00	White Irish - ethnic category 2001 census	White
9i2..00	Other White background - ethnic category 2001 census	White
9i20.00	English - ethnic category 2001 census	White
9i21.00	Scottish - ethnic category 2001 census	White
9i22.00	Welsh - ethnic category 2001 census	White

9i23.00	Cornish - ethnic category 2001 census	White
9i24.00	Northern Irish - ethnic category 2001 census	White
9i25.00	Ulster Scots - ethnic category 2001 census	White
9i26.00	Cypriot (part not stated) - ethnic category 2001 census	White
9i27.00	Greek - ethnic category 2001 census	White
9i28.00	Greek Cypriot - ethnic category 2001 census	White
9i29.00	Turkish - ethnic category 2001 census	White
9i2A.00	Turkish Cypriot - ethnic category 2001 census	White
9i2B.00	Italian - ethnic category 2001 census	White
9i2C.00	Irish Traveller - ethnic category 2001 census	White
9i2D.00	Traveller - ethnic category 2001 census	White
9i2E.00	Gypsy/Romany - ethnic category 2001 census	White
9i2F.00	Polish - ethnic category 2001 census	White
9i2G.00	Baltic Estonian/Latvian/Lithuanian - ethn categ 2001 census	White
9i2H.00	Commonwealth (Russian) Indep States - ethn categ 2001 census	White
9i2J.00	Kosovan - ethnic category 2001 census	White
9i2K.00	Albanian - ethnic category 2001 census	White
9i2L.00	Bosnian - ethnic category 2001 census	White
9i2M.00	Croatian - ethnic category 2001 census	White
9i2N.00	Serbian - ethnic category 2001 census	White
9i2P.00	Other republics former Yugoslavia - ethnic categ 2001 census	White
9i2Q.00	Mixed Irish and other White - ethnic category 2001 census	White
9i2R.00	Oth White European/European unsp/Mixed European 2001 census	White
9i2S.00	Other mixed White - ethnic category 2001 census	White
9i2T.00	Other White or White unspecified ethnic category 2001 census	White
9i3..00	White and Black Caribbean - ethnic category 2001 census	Mixed
9i4..00	White and Black African - ethnic category 2001 census	Mixed
9i5..00	White and Asian - ethnic category 2001 census	Mixed
9i6..00	Other Mixed background - ethnic category 2001 census	Mixed
9i60.00	Black and Asian - ethnic category 2001 census	Mixed
9i61.00	Black and Chinese - ethnic category 2001 census	Mixed
9i62.00	Black and White - ethnic category 2001 census	Mixed
9i63.00	Chinese and White - ethnic category 2001 census	Mixed
9i64.00	Asian and Chinese - ethnic category 2001 census	Mixed
9i65.00	Other Mixed or Mixed unspecified ethnic category 2001 census	Mixed
9i7..00	Indian or British Indian - ethnic category 2001 census	S.Asian
9i8..00	Pakistani or British Pakistani - ethnic category 2001 census	S.Asian
9i9..00	Bangladeshi or British Bangladeshi - ethn categ 2001 census	S.Asian
9iA..00	Other Asian background - ethnic category 2001 census	S.Asian
9iA1.00	Punjabi - ethnic category 2001 census	S.Asian
9iA2.00	Kashmiri - ethnic category 2001 census	S.Asian
9iA3.00	East African Asian - ethnic category 2001 census	S.Asian
9iA4.00	Sri Lankan - ethnic category 2001 census	Other ethnic group
9iA5.00	Tamil - ethnic category 2001 census	Other ethnic group
9iA6.00	Sinhalese - ethnic category 2001 census	Other ethnic group

9iA7.00	Caribbean Asian - ethnic category 2001 census	Mixed
9iA8.00	British Asian - ethnic category 2001 census	S.Asian
9iA9.00	Mixed Asian - ethnic category 2001 census	S.Asian
9iAA.00	Other Asian or Asian unspecified ethnic category 2001 census	S.Asian
9iB..00	Caribbean - ethnic category 2001 census	Black
9iC..00	African - ethnic category 2001 census	Black
9iD..00	Other Black background - ethnic category 2001 census	Black
9iD0.00	Somali - ethnic category 2001 census	Black
9iD1.00	Nigerian - ethnic category 2001 census	Black
9iD2.00	Black British - ethnic category 2001 census	Black
9iD3.00	Mixed Black - ethnic category 2001 census	Black
9iD4.00	Other Black or Black unspecified ethnic category 2001 census	Black
9iE..00	Chinese - ethnic category 2001 census	Other ethnic group
9iF..00	Other - ethnic category 2001 census	Other ethnic group
9iF0.00	Vietnamese - ethnic category 2001 census	Other ethnic group
9iF1.00	Japanese - ethnic category 2001 census	Other ethnic group
9iF2.00	Filipino - ethnic category 2001 census	Other ethnic group
9iF3.00	Malaysian - ethnic category 2001 census	Other ethnic group
9iF4.00	Buddhist - ethnic category 2001 census	Other ethnic group
9iF5.00	Hindu - ethnic category 2001 census	Other ethnic group
9iF6.00	Jewish - ethnic category 2001 census	Other ethnic group
9iF7.00	Muslim - ethnic category 2001 census	Other ethnic group
9iF8.00	Sikh - ethnic category 2001 census	Other ethnic group
9iF9.00	Arab - ethnic category 2001 census	Other ethnic group
9iFA.00	North African - ethnic category 2001 census	Other ethnic group
9iFB.00	Mid East (excl Israeli, Iranian & Arab) - eth cat 2001 cens	Other ethnic group
9iFC.00	Israeli - ethnic category 2001 census	Other ethnic group
9iFD.00	Iranian - ethnic category 2001 census	Other ethnic group
9iFE.00	Kurdish - ethnic category 2001 census	Other ethnic group
9iFF.00	Moroccan - ethnic category 2001 census	Other ethnic group
9iFG.00	Latin American - ethnic category 2001 census	Other ethnic group
9iFH.00	South and Central American - ethnic category 2001 census	Other ethnic group
9iFJ.00	Mauritian/Seychellois/Maldivian/St Helena eth cat 2001census	Other ethnic group
9iFK.00	Any other group - ethnic category 2001 census	Other ethnic group
9iG..00	Ethnic category not stated - 2001 census	Ethnic group not specified
9S...00	Ethnic groups (1991 census)	Ethnic group not specified
9S1..00	White	White
9S10.00	White British	White
9S11.00	White Irish	White
9S12.00	Other white ethnic group	White
9S13.00	White Scottish	White
9S14.00	Other white British ethnic group	White
9S2..00	Black Caribbean	Black
9S3..00	Black African	Black
9S4..00	Black, other, non-mixed origin	Black
9S41.00	Black British	Black
9S42.00	Black Caribbean/W.I./Guyana	Black
9S42.11	Black Caribbean	Black
9S42.12	Black West Indian	Black
9S42.13	Black Guyana	Black

9S43.00	Black N African/Arab/Iranian	Black
9S43.11	Black North African	Black
9S43.12	Black Arab	Black
9S43.13	Black Iranian	Black
9S44.00	Black - other African country	Black
9S45.00	Black E Afric Asia/Indo-Caribb	Mixed
9S45.11	Black East African Asian	Mixed
9S45.12	Black Indo-Caribbean	Mixed
9S46.00	Black Indian sub-continent	Mixed
9S47.00	Black - other Asian	Mixed
9S48.00	Black Black - other	Black
9S5..00	Black - other, mixed	Black
9S51.00	Other Black - Black/White orig	Mixed
9S52.00	Other Black - Black/Asian orig	Mixed
9S6..00	Indian	S.Asian
9S7..00	Pakistani	S.Asian
9S8..00	Bangladeshi	S.Asian
9S9..00	Chinese	Other ethnic group
9SA..00	Other ethnic non-mixed (NMO)	Other ethnic group
9SA1.00	Brit. ethnic minor. spec.(NMO)	Other ethnic group
9SA2.00	Brit. ethnic minor. unsp (NMO)	Other ethnic group
9SA3.00	Caribbean I./W.I./Guyana (NMO)	Black
9SA3.11	Caribbean Island (NMO)	Black
9SA3.12	West Indian (NMO)	Black
9SA3.13	Guyana (NMO)	Black
9SA4.00	N African Arab/Iranian (NMO)	Other ethnic group
9SA4.11	North African Arab (NMO)	Other ethnic group
9SA4.12	Iranian (NMO)	Other ethnic group
9SA5.00	Other African countries (NMO)	Black
9SA6.00	E Afric Asian/Indo-Carib (NMO)	Mixed
9SA6.11	East African Asian (NMO)	S.Asian
9SA7.00	Indian sub-continent (NMO)	S.Asian
9SA8.00	Other Asian (NMO)	S.Asian
9SA9.00	Irish (NMO)	White
9SAA.00	Greek/Greek Cypriot (NMO)	White
9SAA.11	Greek (NMO)	White
9SAA.12	Greek Cypriot (NMO)	White
9SAB.00	Turkish/Turkish Cypriot (NMO)	White
9SAB.11	Turkish (NMO)	White
9SAB.12	Turkish Cypriot (NMO)	White
9SAC.00	Other European (NMO)	White
9SAD.00	Other ethnic NEC (NMO)	Other ethnic group
9SB..00	Other ethnic, mixed origin	Mixed
9SB1.00	Other ethnic, Black/White orig	Mixed
9SB2.00	Other ethnic, Asian/White orig	Mixed
9SB3.00	Other ethnic, mixed white orig	White
9SB4.00	Other ethnic, other mixed orig	Mixed
9SB5.00	Black Caribbean and White	Mixed
9SB6.00	Black African and White	Mixed
9SC..00	Vietnamese	Other ethnic group
9SD..00	Ethnic group not given - patient refused	Ethnic group not specified
9SE..00	Ethnic group not recorded	Ethnic group not specified
9SG..00	Other black ethnic group	Black

9SH..00	Other Asian ethnic group	S.Asian
9SI..00	Irish traveller	White
9SJ..00	Other ethnic group	Other ethnic group
9SZ..00	Ethnic groups (census) NOS	Ethnic group not specified
9T...00	Ethnicity and other related nationality data	Ethnic group not specified
9t0..00	Ethnic category - 2011 census England and Wales	Ethnic group not specified
9t00.00	White:Eng/Welsh/Scot/NI/Brit - England and Wales 2011 census	White
9t01.00	White: Irish - England and Wales ethnic category 2011 census	White
9t02.00	White: Gypsy/Irish Traveller - Eng+Wales eth cat 2011 census	White
9t03.00	White: other White backgrd- Eng+Wales ethnic cat 2011 census	White
9t04.00	Mixed: White+Black Caribbean - Eng+Wales eth cat 2011 census	Mixed
9t05.00	Mixed: White+Black African - Eng+Wales eth cat 2011 census	Mixed
9t06.00	Mixed: White+Asian - Eng+Wales ethnic category 2011 census	Mixed
9t08.00	Asian/Asian Brit: Indian - Eng+Wales ethnic cat 2011 census	S.Asian
9t09.00	Asian/Asian British:Pakistani- Eng+Wales eth cat 2011 census	S.Asian
9t0A.00	Asian/Asian Brit: Bangladeshi- Eng+Wales eth cat 2011 census	S.Asian
9t0B.00	Asian/Asian Brit: Chinese - Eng+Wales ethnic cat 2011 census	Other ethnic group
9t0C.00	Asian/Asian Brit: other Asian- Eng+Wales eth cat 2011 census	S.Asian
9t0D.00	Black/African/Carib/Black Brit: African- Eng+Wales 2011 cens	Black
9t0E.00	Black/African/Caribbn/Black Brit: Caribbean - Eng+Wales 2011	Black
9t0F.00	Black/Afr/Carib/Black Brit: other Black- Eng+Wales 2011 cens	Black
9t0G.00	Other ethnic group: Arab - Eng+Wales ethnic cat 2011 census	Other ethnic group
9t0H.00	Other ethnic: any other grp - Eng+Wales eth cat 2011 census	Other ethnic group
9T1..00	New Zealand ethnic groups	White
9t12.00	Mixed: White and Black Caribbean - NI ethnic cat 2011 census	Mixed
9t13.00	Mixed: White and Black African - NI ethnic cat 2011 census	Mixed
9t14.00	Mixed: White and Asian - NI ethnic category 2011 census	Mixed
9t15.00	Mixed: other Mixed/multiple ethnic backgrd - NI 2011 census	Mixed
9t16.00	Asian or Asian British: Indian - NI ethnic cat 2011 census	S.Asian
9t17.00	Asian/Asian British: Pakistani - NI ethnic cat 2011 census	S.Asian
9t18.00	Asian/Asian British: Bangladeshi - NI ethnic cat 2011 census	S.Asian
9t19.00	Asian/Asian British: Chinese - NI ethnic cat 2011 census	Other ethnic group
9T1A.00	Other Pacific ethnic group	Other ethnic group
9t1E.00	Other ethnic group: Arab - NI ethnic category 2011 census	Other ethnic group
9t1F.00	Other ethnic group: any other grp- NI ethnic cat 2011 census	Other ethnic group

9T1Y.00	Other New Zealand ethnic group	White
9T1Z.00	New Zealand ethnic group NOS	White
9t2..00	Ethnic category - 2011 census Scotland	Ethnic group not specified
9t20.00	White: Scottish - Scotland ethnic category 2011 census	White
9t21.00	White: other British - Scotland ethnic category 2011 census	White
9t22.00	White: Irish - Scotland ethnic category 2011 census	White
9t24.00	White: Polish - Scotland ethnic category 2011 census	White
9t25.00	White: other White ethnic grp- Scotland ethnic cat 2011 cens	White
9t26.00	Mixed/multiple ethnic grps: any- Scot ethnic cat 2011 census	Mixed
9t27.00	Asian: Pakistani/Pakistani Scot/Pakistani Brit- Scot 2011	S.Asian
9t28.00	Asian: Indian, Indian Scot/Indian Brit- Scotland 2011 census	S.Asian
9t29.00	Bangladeshi, Bangladeshi Scot or Bangladeshi Brit- Scot 2011	S.Asian
9t2A.00	Asian: Chinese - Scotland ethnic category 2011 census	Other ethnic group
9t2B.00	Asian: other Asian group - Scotland ethnic cat 2011 census	S.Asian
9t2C.00	African: African/African Scot/African Brit - Scotland 2011	Black
9t2D.00	African: any other African - Scotland ethnic cat 2011 census	Black
9t2G.00	Carib/Black: any other Black/Caribbean grp - Scotland 2011	Black
9t2H.00	Other ethnic grp: Arab/Arab Scot/Arab British- Scotland 2011	Other ethnic group
9t2J.00	Other ethnic grp: any other ethnic grp- Scotland 2011 census	Other ethnic group
**Free-text field from Hospital Episode Statistics' patient file**		
**Free text**	**Ethnic group**	
Bangladeshi	S.Asian	
Bl_Afric	Black	
Bl_Carib	Black	
Bl_Other	Black	
Chinese	Other ethnic group	
Indian	S.Asian	
Mixed	Mixed	
Oth_Asian	S.Asian	
Other	Other ethnic group	
Pakistani	S.Asian	
Unknown	Ethnic group not specified	
White	White	
**Primary care Read codes for severe health needs** (identified from Tests, Referrals or Clinical files)		
**Read Code**	**Read Code Description**	**Interpretation**
2822.00	O/E - grand mal fit	Epilepsy
2826.00	O/E - salaam attack	Epilepsy
6671.00	Initial epilepsy assessment	Epilepsy
6672.00	Follow-up epilepsy assessment	Epilepsy
6674.00	Epilepsy associated problems	Epilepsy
6677.00	Epilepsy drug side effects	Epilepsy
6678.00	Epilepsy treatment changed	Epilepsy

6679.00	Epilepsy treatment started	Epilepsy
14On.00	At risk of sudden unexpected death in epilepsy	Epilepsy
1B1W.00	Transient epileptic amnesia	Epilepsy
1B26.00	Trigger factor for seizure	Epilepsy
1O30.00	Epilepsy confirmed	Epilepsy
44W4.11	Epilim: blood level	Epilepsy
667..00	Epilepsy monitoring	Epilepsy
667B.00	Nocturnal epilepsy	Epilepsy
667D.00	Epilepsy control poor	Epilepsy
667E.00	Epilepsy care arrangement	Epilepsy
667G.00	Epilepsy restricts employment	Epilepsy
667H.00	Epilepsy prevents employment	Epilepsy
667J.00	Epilepsy impairs education	Epilepsy
667K.00	Epilepsy limits activities	Epilepsy
667M.00	Epilepsy management plan given	Epilepsy
667N.00	Epilepsy severity	Epilepsy
667R.00	2 to 4 seizures a month	Epilepsy
667S.00	1 to 7 seizures a week	Epilepsy
667T.00	Daily seizures	Epilepsy
667V.00	Many seizures a day	Epilepsy
667W.00	Emergency epilepsy treatment since last appointment	Epilepsy
667Z.00	Epilepsy monitoring NOS	Epilepsy
9Of3.00	Epilepsy monitoring verbal invite	Epilepsy
9Of4.00	Epilepsy monitoring telephone invite	Epilepsy
9Of5.00	Epilepsy monitoring call first letter	Epilepsy
9Of6.00	Epilepsy monitoring call second letter	Epilepsy
9Of7.00	Epilepsy monitoring call third letter	Epilepsy
Eu05212	[X]Schizophrenia-like psychosis in epilepsy	Epilepsy
Eu05y11	[X]Epileptic psychosis NOS	Epilepsy
Eu06013	[X]Limbic epilepsy personality	Epilepsy
Eu80300	[X]Acquired aphasia with epilepsy [Landau - Kleffner]	Epilepsy
F132100	Progressive myoclonic epilepsy	Epilepsy
F132z12	Myoclonic seizure	Epilepsy
F25..00	Epilepsy	Epilepsy
F250.00	Generalised nonconvulsive epilepsy	Epilepsy
F250200	Epileptic seizures - atonic	Epilepsy
F250300	Epileptic seizures - akinetic	Epilepsy
F250500	Lennox-Gastaut syndrome	Epilepsy
F250y00	Other specified generalised nonconvulsive epilepsy	Epilepsy
F250z00	Generalised nonconvulsive epilepsy NOS	Epilepsy
F251.00	Generalised convulsive epilepsy	Epilepsy
F251000	Grand mal (major) epilepsy	Epilepsy
F251011	Tonic-clonic epilepsy	Epilepsy
F251100	Neonatal myoclonic epilepsy	Epilepsy
F251111	Otohara syndrome	Epilepsy
F251200	Epileptic seizures - clonic	Epilepsy

F251300	Epileptic seizures - myoclonic	Epilepsy
F251400	Epileptic seizures - tonic	Epilepsy
F251500	Tonic-clonic epilepsy	Epilepsy
F251600	Grand mal seizure	Epilepsy
F251y00	Other specified generalised convulsive epilepsy	Epilepsy
F251z00	Generalised convulsive epilepsy NOS	Epilepsy
F252.00	Petit mal status	Epilepsy
F253.00	Grand mal status	Epilepsy
F253.11	Status epilepticus	Epilepsy
F254.00	Partial epilepsy with impairment of consciousness	Epilepsy
F254000	Temporal lobe epilepsy	Epilepsy
F254100	Psychomotor epilepsy	Epilepsy
F254200	Psychosensory epilepsy	Epilepsy
F254300	Limbic system epilepsy	Epilepsy
F254400	Epileptic automatism	Epilepsy
F254500	Complex partial epileptic seizure	Epilepsy
F254z00	Partial epilepsy with impairment of consciousness NOS	Epilepsy
F255.00	Partial epilepsy without impairment of consciousness	Epilepsy
F255000	Jacksonian, focal or motor epilepsy	Epilepsy
F255011	Focal epilepsy	Epilepsy
F255012	Motor epilepsy	Epilepsy
F255100	Sensory induced epilepsy	Epilepsy
F255200	Somatosensory epilepsy	Epilepsy
F255300	Visceral reflex epilepsy	Epilepsy
F255311	Partial epilepsy with autonomic symptoms	Epilepsy
F255400	Visual reflex epilepsy	Epilepsy
F255500	Unilateral epilepsy	Epilepsy
F255600	Simple partial epileptic seizure	Epilepsy
F255y00	Partial epilepsy without impairment of consciousness OS	Epilepsy
F255z00	Partial epilepsy without impairment of consciousness NOS	Epilepsy
F256.11	Lightning spasms	Epilepsy
F256.12	West syndrome	Epilepsy
F256000	Hypsarrhythmia	Epilepsy
F256100	Salaam attacks	Epilepsy
F257.00	Kojevnikov's epilepsy	Epilepsy
F258.00	Post-ictal state	Epilepsy
F259.00	Early infant epileptic encephalopathy wth suppression bursts	Epilepsy
F259.11	Ohtahara syndrome	Epilepsy
F25A.00	Juvenile myoclonic epilepsy	Epilepsy
F25F.00	Photosensitive epilepsy	Epilepsy
F25G.00	Severe myoclonic epilepsy in infancy	Epilepsy
F25H.00	Generalised seizure	Epilepsy
F25X.00	Status epilepticus, unspecified	Epilepsy
F25y.00	Other forms of epilepsy	Epilepsy
F25y000	Cursive (running) epilepsy	Epilepsy

F25y100	Gelastic epilepsy	Epilepsy
F25y200	Locl-rlt(foc)(part)idiop epilep&epilptic syn seiz locl onset	Epilepsy
F25y300	Complex partial status epilepticus	Epilepsy
F25y500	Panayiotopoulos syndrome	Epilepsy
F25yz00	Other forms of epilepsy NOS	Epilepsy
F25z.00	Epilepsy NOS	Epilepsy
F25z.11	Fit (in known epileptic) NOS	Epilepsy
Fyu5000	[X]Other generalized epilepsy and epileptic syndromes	Epilepsy
Fyu5100	[X]Other epilepsy	Epilepsy
Fyu5200	[X]Other status epilepticus	Epilepsy
Fyu5900	[X]Status epilepticus, unspecified	Epilepsy
PK5..12	Epiloia	Epilepsy
R003400	[D]Nocturnal seizure	Epilepsy
SC20000	Traumatic epilepsy	Epilepsy
ZS82.00	Acquired epileptic aphasia	Epilepsy
3930.00	Bowels: incontinent	Incontinence
3931.00	Bowels: occasional accident	Incontinence
3940.00	Bladder: incontinent	Incontinence
3941.00	Bladder: occasional accident	Incontinence
16F..00	Double incontinence	Incontinence
19E2.00	Soiling - encopresis	Incontinence
19E2.11	Encopresis symptom	Incontinence
19E2.12	Soiling symptom	Incontinence
19E3.00	Incontinent of faeces	Incontinence
19E3.11	Incontinent of faeces symptom	Incontinence
1A22.00	Enuresis	Incontinence
1A22000	Nocturnal enuresis	Incontinence
1A22011	Bedwetting	Incontinence
1A22100	Daytime enuresis	Incontinence
1A23.00	Incontinence of urine	Incontinence
1A24.00	Stress incontinence	Incontinence
1A24.11	Stress incontinence - symptom	Incontinence
1A26.00	Urge incontinence of urine	Incontinence
7B33800	Insertion retropubic device stress urinary incontinence NEC	Incontinence
7B33C00	Insertion retropubic dev fem stress urinary incontinence NEC	Incontinence
7B42111	Insertion of Kaufman prosthesis for male incontinence	Incontinence
7B42113	Insertion of Rosen prosthesis for male incontinence	Incontinence
8C14.00	Incontinence care	Incontinence
8D7..12	Incontinence control	Incontinence
8D71.00	Incontinence control	Incontinence
8D73.00	Nocturnal bladder warning syst	Incontinence
8D73.11	Enuretic alarm	Incontinence
8D73.12	Enuresis alarm	Incontinence
8HTt.00	Referral to enuresis clinic	Incontinence
8HTX.00	Referral to incontinence clinic	Incontinence

8O6..00	Enuresis support	Incontinence
9No6.00	Seen in enuresis clinic	Incontinence
E276.00	Non-organic enuresis	Incontinence
E276000	Non-organic primary enuresis	Incontinence
E276100	Non-organic secondary enuresis	Incontinence
E276z00	Non-organic enuresis NOS	Incontinence
E277.00	Non-organic encopresis	Incontinence
E277000	Non-organic continuous encopresis	Incontinence
E277z00	Non-organic encopresis NOS	Incontinence
Eu9y000	[X]Nonorganic enuresis	Incontinence
Eu9y100	[X]Nonorganic encopresis	Incontinence
F246112	Neurogenic bladder	Incontinence
K16V011	Neurogenic bladder	Incontinence
K198.00	Stress incontinence	Incontinence
K586.00	Stress incontinence - female	Incontinence
Kyu5A00	[X]Other specified urinary incontinence	Incontinence
M129500	Incontinence-associated dermatitis	Incontinence
R076.00	[D]Incontinence of faeces	Incontinence
R076000	[D]Encopresis NOS	Incontinence
R076100	[D]Sphincter ani incontinence	Incontinence
R076z00	[D]Incontinence of faeces NOS	Incontinence
R083.00	[D]Incontinence of urine	Incontinence
R083000	[D]Enuresis NOS	Incontinence
R083100	[D]Urethral sphincter incontinence	Incontinence
R083200	[D] Urge incontinence	Incontinence
R083z00	[D]Incontinence of urine NOS	Incontinence
Z1J..00	Procedures to aid continence	Incontinence
Z9EA.00	Provision of incontinence appliance	Incontinence
Z9EA100	Provision of nocturnal bladder warning system	Incontinence
Z9EA111	Provision of enuresis alarm	Incontinence
Z9EA112	Provision of enuretic alarm	Incontinence
Z9MO.00	Enuresis support	Incontinence
ZQ3C.00	Bowels incontinence assessment	Incontinence
6688.00	Registered partially sighted	Severe visual impairment
6688.11	Registered partially blind	Severe visual impairment
6689.00	Registered blind	Severe visual impairment
6689.11	Registered severely sight impaired	Severe visual impairment
6689.11	Registered severely sight impaired	Severe visual impairment
7202.00	Insertion of prosthesis of eye	Severe visual impairment
7203.00	Attention to prosthesis of eye	Severe visual impairment
7202100	Insertion of prosthetic replacement for eyeball	Severe visual impairment
7203000	Revision of prosthetic replacement for orbit	Severe visual impairment
7203100	Revision of prosthetic replacement for eyeball	Severe visual impairment
7203300	Removal of prosthetic replacement for eyeball	Severe visual impairment
14T1.00	H/O: artificial eyeglobe	Severe visual impairment
1a00000	Uses guide dog for the blind	Severe visual impairment

1B75.00	Loss of vision	Severe visual impairment
22E6.00	O/E - glass (prosthetic) eye	Severe visual impairment
22E6.11	O/E - false eye	Severe visual impairment
22E6.12	O/E - glass eye	Severe visual impairment
22E6.13	O/E - prosthetic eye	Severe visual impairment
22EF.00	O/E - has one eye	Severe visual impairment
2B69.00	O/E -R-eye counts fingers only	Severe visual impairment
2B6A.00	O/E-R-eye perceives light only	Severe visual impairment
2B6A.11	O/E - blind R-eye	Severe visual impairment
2B6B.00	O/E - R-eye completely blind	Severe visual impairment
2B6C.00	O/E - R-eye sees hand movements	Severe visual impairment
2B6P.00	O/E - pinhole R-eye sees hand movements	Severe visual impairment
2B6Q.00	O/E - pinhole R-eye counts fingers only	Severe visual impairment
2B6R.00	O/E - pinhole R-eye perceives light only	Severe visual impairment
2B6S.00	O/E - pinhole R-eye completely blind	Severe visual impairment
2B6T.00	O/E - R-eye visual acuity (corrected) 1/60	Severe visual impairment
2B6V.00	O/E - R-eye visual acuity (corrected) 2/60	Severe visual impairment
2B6W.00	O/E - R-eye visual acuity (corrected) 4/60	Severe visual impairment
2B6X.00	O/E - R-eye visual acuity (corrected) 5/60	Severe visual impairment
2B79.00	O/E -L-eye counts fingers only	Severe visual impairment
2B7A.00	O/E-L-eye perceives light only	Severe visual impairment
2B7A.11	O/E - blind L-eye	Severe visual impairment
2B7B.00	O/E - L-eye completely blind	Severe visual impairment
2B7C.00	O/E - L-eye sees hand movements	Severe visual impairment
2B7P.00	O/E - pinhole L-eye sees hand movements	Severe visual impairment
2B7Q.00	O/E - pinhole L-eye counts fingers only	Severe visual impairment
2B7R.00	O/E - pinhole L-eye perceives light only	Severe visual impairment
2B7S.00	O/E - pinhole L-eye completely blind	Severe visual impairment
2B7T.00	O/E - L-eye visual acuity (corrected) 1/60	Severe visual impairment
2B7V.00	O/E - L-eye visual acuity (corrected) 2/60	Severe visual impairment
2B7W.00	O/E - L-eye visual acuity (corrected) 4/60	Severe visual impairment
2B7X.00	O/E - L-eye visual acuity (corrected) 5/60	Severe visual impairment
2BBr.00	Impaired vision due to diabetic retinopathy	Severe visual impairment
668B.00	Poor visual acuity	Severe visual impairment
668C.00	Certificate of vision impairment	Severe visual impairment
668D.00	Registered sight impaired	Severe visual impairment
7202y00	Other specified insertion of prosthesis of eye	Severe visual impairment
7202z00	Insertion of prosthesis of eye NOS	Severe visual impairment
7203y00	Other specified attention to prosthesis of eye	Severe visual impairment
7203z00	Attention to prosthesis of eye NOS	Severe visual impairment
8D3..00	Visual aid	Severe visual impairment
8D3..13	Visual aid provision	Severe visual impairment
8D31.00	Physiolog. visual assistance	Severe visual impairment
8D36.00	Removable artificial eye	Severe visual impairment
8D3Z.00	Visual aid NOS	Severe visual impairment
8E1..00	Visual defect-remedial therapy	Severe visual impairment

8E1Z.00	Visual defect-remed.therap.NOS	Severe visual impairment
8F6..11	Blind rehabilitation	Severe visual impairment
8F61.00	Blind rehabilitation	Severe visual impairment
8F62.00	Blind lead dog rehabilitation	Severe visual impairment
8HlE.00	Referral to visual impairment multidisciplinary team	Severe visual impairment
8O0D.00	Provision of support as Braille user	Severe visual impairment
9m08.00	Excluded from diabetic retinopathy screening as blind	Severe visual impairment
9Nfa.00	Requires deafblind manual alphabet interpreter	Severe visual impairment
9NfB.00	Requires deafblind communicator guide	Severe visual impairment
9Nfb.00	Requires deafblind block alphabet interpreter	Severe visual impairment
9NfM.00	Requires information in contracted (Grade 2) Braille	Severe visual impairment
9NfN.00	Requires information in uncontracted (Grade 1) Braille	Severe visual impairment
9NlD.00	Seen by visual impairment teacher	Severe visual impairment
9NnR.00	Visual frame sign language interpreter needed	Severe visual impairment
F101200	Spielmeyer-Vogt (Batten) disease	Severe visual impairment
F404100	Blind hypotensive eye	Severe visual impairment
F404200	Blind hypertensive eye	Severe visual impairment
F49..00	Blindness and low vision	Severe visual impairment
F49..11	Impaired vision	Severe visual impairment
F49..12	Low vision	Severe visual impairment
F49..13	Partial sight	Severe visual impairment
F49..14	Sight impaired	Severe visual impairment
F490.00	Blindness, both eyes	Severe visual impairment
F490000	Unspecified blindness both eyes	Severe visual impairment
F490100	Both eyes total visual impairment	Severe visual impairment
F490200	Better eye: near total VI, Lesser eye: unspecified	Severe visual impairment
F490400	Better eye: near total VI, Lesser eye: near total VI	Severe visual impairment
F490600	Better eye: profound VI, Lesser eye: total VI	Severe visual impairment
F490900	Acquired blindness, both eyes	Severe visual impairment
F490z00	Blindness both eyes NOS	Severe visual impairment
F491.00	Better eye: low vision, Lesser eye: profound VI	Severe visual impairment
F491000	One eye blind, one eye low vision	Severe visual impairment
F491100	Better eye: severe VI, Lesser eye: blind, unspecified	Severe visual impairment
F491300	Better eye: severe VI, Lesser eye: near total VI	Severe visual impairment
F491400	Better eye: severe VI, Lesser eye: profound VI	Severe visual impairment
F491500	Better eye: moderate VI, Lesser eye: blind, unspecified	Severe visual impairment
F491700	Better eye: moderate VI, Lesser eye: near total VI	Severe visual impairment
F491z00	One eye blind, one eye low vision NOS	Severe visual impairment
F492.00	Low vision, both eyes	Severe visual impairment
F492000	Low vision, both eyes unspecified	Severe visual impairment
F492200	Better eye: severe VI, Lesser eye: severe VI	Severe visual impairment
F492300	Better eye: moderate VI, Lesser eye: low vision unspecified	Severe visual impairment
F492400	Better eye: moderate VI, Lesser eye: severe VI	Severe visual impairment
F492500	Better eye: moderate VI, Lesser eye: moderate VI	Severe visual impairment
F492z00	Low vision, both eyes NOS	Severe visual impairment

F493.00	Visual loss, both eyes unqualified	Severe visual impairment
F494.00	Legal blindness USA	Severe visual impairment
F495.00	Profound impairment, one eye	Severe visual impairment
F495000	Blindness, one eye, unspecified	Severe visual impairment
F495100	Lesser eye: total visual impairment, Better eye: unspecified	Severe visual impairment
F495200	Lesser eye: total VI, Better eye: near normal vision	Severe visual impairment
F495300	Lesser eye: total VI, Better eye: normal vision	Severe visual impairment
F495400	Lesser eye: near total VI, Better eye: unspecified	Severe visual impairment
F495500	Lesser eye: near total VI, Better eye: near normal vision	Severe visual impairment
F495600	Lesser eye: near total VI, Better eye: normal vision	Severe visual impairment
F495800	Lesser eye: profound VI, Better eye: near normal vision	Severe visual impairment
F495A00	Acquired blindness, one eye	Severe visual impairment
F495z00	Profound impairment one eye NOS	Severe visual impairment
F496.00	Low vision, one eye	Severe visual impairment
F496000	Low vision, one eye, unspecified	Severe visual impairment
F496100	Lesser eye: severe VI, Better eye: unspecified	Severe visual impairment
F496200	Lesser eye: severe VI, Better eye: near normal vision	Severe visual impairment
F496300	Lesser eye: severe VI, Better eye: normal vision	Severe visual impairment
F496400	Lesser eye: moderate VI, Better eye: unspecified	Severe visual impairment
F496500	Lesser eye: moderate VI, Better eye: near normal vision	Severe visual impairment
F496600	Lesser eye: moderate VI, Better eye: normal vision	Severe visual impairment
F496z00	Low vision, one eye NOS	Severe visual impairment
F497.00	Severe visual impairment, binocular	Severe visual impairment
F498.00	Moderate visual impairment, binocular	Severe visual impairment
F49A.00	Blindness, monocular	Severe visual impairment
F49B.00	Severe visual impairment, monocular	Severe visual impairment
F49C.00	Moderate visual impairment, monocular	Severe visual impairment
F49D.00	Visual impairment	Severe visual impairment
F49y.00	Visual loss, one eye, unqualified	Severe visual impairment
F49z.00	Visual loss NOS	Severe visual impairment
F49z.11	Acquired blindness	Severe visual impairment
F49z000	Charles Bonnet syndrome	Severe visual impairment
F4H..11	Visual path disorder	Severe visual impairment
F4H6.00	Other visual pathway disorder	Severe visual impairment
F4H6100	Visual pathway disorder due to vascular disorder	Severe visual impairment
F4H6z00	Other visual pathway disorder NOS	Severe visual impairment
F4H7.00	Visual cortex disorder	Severe visual impairment
F4H7100	Visual cortex disorder due to vascular disorder	Severe visual impairment
F4H7300	Cortical blindness	Severe visual impairment
F4H7z00	Visual cortex disorder NOS	Severe visual impairment
F4Hz.00	Disorder of optic nerve or visual pathway NOS	Severe visual impairment
Fy1..00	Combined visual and hearing impairment	Severe visual impairment
Fy1..12	Deafblind	Severe visual impairment
FyuJ.00	[X]Disorders of optic nerve and visual pathway	Severe visual impairment
FyuL.00	[X]Visual disturbances and blindness	Severe visual impairment

FyuL100	[X]Other visual disturbances	Severe visual impairment
SJ0..00	Optic nerve and pathway injury	Severe visual impairment
SJ00.00	Optic nerve (2nd) injury	Severe visual impairment
SJ01.00	Optic chiasm injury	Severe visual impairment
SJ02.00	Optic pathway injury	Severe visual impairment
SJ03.00	Visual cortex injury	Severe visual impairment
SJ0z.00	Optic nerve or pathway injury NOS	Severe visual impairment
SJ0z.11	Blindness - traumatic - NOS	Severe visual impairment
Z96..00	Provision for visual and hearing impairment	Severe visual impairment
Z961.00	Provision of guide help for visual and hearing impairment	Severe visual impairment
Z962.00	Provision of communicator for visual and hearing impairment	Severe visual impairment
Z9E2.00	Optical low vision aid provision	Severe visual impairment
Z9E3.00	Provision of optical low vision aid - near	Severe visual impairment
Z9E3100	Provision of magnifier low vision aid - near	Severe visual impairment
Z9E3200	Provision of low vision hand magnifier	Severe visual impairment
Z9E3300	Provision of low vision stand magnifier	Severe visual impairment
Z9E3500	Provision of spectacle low vision aid - near	Severe visual impairment
Z9E3600	Provision of telescopic spectacles	Severe visual impairment
Z9E3700	Provision of spectacle magnifier	Severe visual impairment
Z9E3900	Near low vision aid - clip-on spectacle magnifier	Severe visual impairment
Z9E3A00	Provision of spectacle telescope	Severe visual impairment
Z9E3B00	Near low vision aid - integral spectacle telescope	Severe visual impairment
Z9E3C00	Near low vision aid - clip-on spectacle telescope	Severe visual impairment
Z9E3D00	Near low vision aid - extra cap for telescope	Severe visual impairment
Z9E3E00	Provision of headband telescope	Severe visual impairment
Z9E4.00	Provision of optical low vision aid - distance	Severe visual impairment
Z9E5.00	Provision of non-optical low vision aid	Severe visual impairment
Z9E5200	Provision of closed circuit television	Severe visual impairment
Z9E5300	Provision of image intensifier	Severe visual impairment
Z9E5400	Provision of ancillary low vision aid	Severe visual impairment
Z9E5700	Provision of work board	Severe visual impairment
Z9E6.00	Provision of visual appliance	Severe visual impairment
Z9E6500	Provision of audiotaped services	Severe visual impairment
Z9E6600	Provision of talking book	Severe visual impairment
ZN56800	Blind telephone user	Severe visual impairment
ZV410	[V]Problems with sight	Severe visual impairment
ZV52200	[V]Fitting or adjustment of artificial eye	Severe visual impairment
7007300	Insertion of auditory implant to brainstem	Severe hearing impairment
7308400	Placement of hearing implant in external ear	Severe hearing impairment
7308500	Attention to hearing implant in external ear	Severe hearing impairment
7308600	Removal of hearing implant from external ear	Severe hearing impairment
7311A00	Insertn bone anchors subcutaneous bone anchored hearing aid	Severe hearing impairment
7317E00	Removal of hearing implant from middle ear	Severe hearing impairment
7319000	Insertion fixtures bone anchored hearing prosthesis Stage 1	Severe hearing impairment

7319100	Insertion fixtures bone anchored hearing prosthesis Stage 2	Severe hearing impairment
7319200	Reduction soft tissue for bone anchored hearing prosthesis	Severe hearing impairment
7319300	Attention to fixtures for bone anchored hearing prosthesis	Severe hearing impairment
7319400	One stage insert fixtures bone anchored hearing prosthesis	Severe hearing impairment
7319500	Fitting external hearing prosthesis bone anchored fixtures	Severe hearing impairment
1C13.00	Deafness	Severe hearing impairment
1C13300	Bilateral deafness	Severe hearing impairment
2BL..11	O/E - deaf	Severe hearing impairment
2BL3.00	O/E - significantly deaf	Severe hearing impairment
2BL4.00	O/E - very deaf	Severe hearing impairment
2BL5.00	O/E - completely deaf	Severe hearing impairment
2DG..00	Hearing aid worn	Severe hearing impairment
2DH0.00	Uses hearing loop	Severe hearing impairment
7317C00	Placement of hearing implant in middle ear	Severe hearing impairment
7317D00	Attention to hearing implant in middle ear	Severe hearing impairment
7319.00	Attachment of bone anchored hearing prosthesis	Severe hearing impairment
7319y00	Other specified attachment bone anchored hearing prosthesis	Severe hearing impairment
7319z00	Attachment of bone anchored hearing prosthesis NOS	Severe hearing impairment
8D2..00	Auditory aid	Severe hearing impairment
8D2..11	Auditory aid provision	Severe hearing impairment
8D2..12	Hearing aid provision	Severe hearing impairment
8D21.00	Provide head worn hearing aid	Severe hearing impairment
8D22.00	Provide body worn hearing aid	Severe hearing impairment
8D23.00	Ear fitting hearing aid	Severe hearing impairment
8D24.00	Replace hearing aid battery	Severe hearing impairment
8D25.00	Physiolog. hearing assistance	Severe hearing impairment
8D2Z.00	Auditory aid NOS	Severe hearing impairment
8E3..00	Deafness remedial therapy	Severe hearing impairment
8E3Z.00	Deafness remedial therapy NOS	Severe hearing impairment
8M41.00	Hearing aid requested	Severe hearing impairment
9N0b.00	Seen in hearing aid clinic	Severe hearing impairment
9NfB.00	Requires deafblind communicator guide	Severe hearing impairment
A560200	Rubella deafness	Severe hearing impairment
F581211	Noise induced deafness	Severe hearing impairment
F59..11	Deafness	Severe hearing impairment
F590.11	Conductive deafness	Severe hearing impairment
F591.13	Perceptive deafness	Severe hearing impairment
F591211	Nerve deafness	Severe hearing impairment
F591400	Congenital sensorineural deafness	Severe hearing impairment
F591500	Ototoxicity - deafness	Severe hearing impairment
F591511	Drug ototoxicity - deafness	Severe hearing impairment
F591800	Congenital prelingual deafness	Severe hearing impairment
F592.00	Mixed conductive and sensorineural deafness	Severe hearing impairment
F593.00	Deaf mutism, NEC	Severe hearing impairment

F596.00	Maternally inherited deafness	Severe hearing impairment
F598.00	Moderate acquired hearing loss	Severe hearing impairment
F599.00	Severe acquired hearing loss	Severe hearing impairment
F59A.00	Profound acquired hearing loss	Severe hearing impairment
F59A.11	Deafened	Severe hearing impairment
F59z.00	Deafness NOS	Severe hearing impairment
F59z.11	Chronic deafness	Severe hearing impairment
Fy1..00	Combined visual and hearing impairment	Severe hearing impairment
Fy1..12	Deafblind	Severe hearing impairment
FyuU000	[X]Deaf mutism, not elsewhere classified	Severe hearing impairment
P40z.11	Deafness due to congenital anomaly NEC	Severe hearing impairment
SJ15.12	Deafness - traumatic - NOS	Severe hearing impairment
Z8B5.00	Ability to use hearing aid	Severe hearing impairment
Z8B5100	Able to use hearing aid	Severe hearing impairment
Z8B5200	Unable to use hearing aid	Severe hearing impairment
Z8B5300	Does use hearing aid	Severe hearing impairment
Z8B5311	Uses hearing aid	Severe hearing impairment
Z8B5500	Difficulty using hearing aid	Severe hearing impairment
Z911.00	Hearing aid procedure	Severe hearing impairment
Z911100	Fit hearing aid	Severe hearing impairment
Z911300	Adjust hearing aid settings	Severe hearing impairment
Z911400	Changing hearing aid battery	Severe hearing impairment
Z911500	Checking hearing aid	Severe hearing impairment
Z911700	Switching on hearing aid	Severe hearing impairment
Z911800	Turning off hearing aid	Severe hearing impairment
Z911900	Putting on hearing aid	Severe hearing impairment
ZE87.00	Hearing loss	Severe hearing impairment
ZE87.16	HL - Hearing loss	Severe hearing impairment
Z911A00	Listening for feedback whistle of hearing aid	Severe hearing impairment
Z911B00	Attention to hearing aid	Severe hearing impairment
Z911E00	Fit ear mould for existing hearing aid	Severe hearing impairment
ZE87.00	Hearing loss Severe Hearing impairment	Severe hearing impairment
ZE87.16	HL - Hearing loss Severe Hearing impairment	Severe hearing impairment
Z9E8100	Hearing aid provision	Severe hearing impairment
Z9E8111	Auditory aid provision	Severe hearing impairment
ZE83200	Hearing for loud voice impaired	Severe hearing impairment
ZE84200	Hearing for voice impaired	Severe hearing impairment
ZE87.11	Deafness	Severe hearing impairment
ZE87.13	Hard of hearing	Severe hearing impairment
ZE87.17	HOH - Hard of hearing	Severe hearing impairment
ZN56900	Deaf telephone user	Severe hearing impairment
ZT12711	Voice associated with hearing loss	Severe hearing impairment
ZV45G00	[V]Presence of external hearing-aid	Severe hearing impairment
ZV45N00	[V]Bone anchored hearing aid in situ	Severe hearing impairment
ZV53200	[V]Fitting or adjustment of hearing aid	Severe hearing impairment

ZV53D00	[V]Adjustment and management of implanted hearing device	Severe hearing impairment
7319600	First stge ins fixtures for bone anchored hearing prosthesis	Severe hearing impairment
7319700	Second stage ins fixtures for bone anchored hearing prosth	Severe hearing impairment
13o8M00	Uses sign language	Severe hearing impairment
13ZL.00	Using lip-reading	Severe hearing impairment
13ZM.00	Using British sign language	Severe hearing impairment
13ZP.00	Using Makaton sign language	Severe hearing impairment
2DH1.00	Does use hearing aid	Severe hearing impairment
8D26.00	Provision of replacement hearing aid	Severe hearing impairment
9Nfa.00	Requires deafblind manual alphabet interpreter	Severe hearing impairment
9Nfb.00	Requires deafblind block alphabet interpreter	Severe hearing impairment
9NnR.00	Visual frame sign language interpreter needed	Severe hearing impairment
9NnS.00	Hands-on signing interpreter needed	Severe hearing impairment
F591900	Bilateral profound sensorineural hearing loss	Severe hearing impairment
F591A00	Bilateral congenital sensorineural hearing loss	Severe hearing impairment
F591B00	Profound sensorineural hearing loss	Severe hearing impairment
F591E00	Severe sensorineural hearing loss	Severe hearing impairment
F594.00	High frequency deafness	Severe hearing impairment
F595.00	Low frequency deafness	Severe hearing impairment
Z96..00	Provision for visual and hearing impairment	Severe hearing impairment
Z961.00	Provision of guide help for visual and hearing impairment	Severe hearing impairment
Z962.00	Provision of communicator for visual and hearing impairment	Severe hearing impairment
Z9E3314	Auditory aid provision	Severe hearing impairment
Z9E8.00	Provision of auditory appliance	Severe hearing impairment
ZE83300	Unable to hear loud voice	Severe hearing impairment
ZN56A00	Deaf-blind telephone user	Severe hearing impairment
ZV53011	[V]Fitting or adjustment of auditory substitution device	Severe hearing impairment
2835.00	O/E - paraplegia	Severe mobility difficulties
2836.00	O/E - quadriplegia	Severe mobility difficulties
3960.00	Dependent: chair/bed transfer	Severe mobility difficulties
3961.00	Able to sit: chair/bed trans.	Severe mobility difficulties
3980.00	Immobile	Severe mobility difficulties
3981.00	Independent in wheelchair	Severe mobility difficulties
3982.00	Minimal help in wheelchair	Severe mobility difficulties
3990.00	Unable to climb stairs	Severe mobility difficulties
13C5.00	Confined to chair	Severe mobility difficulties
13C5.11	Chairbound	Severe mobility difficulties
13C6.00	Bed-ridden	Severe mobility difficulties
13C6.11	Bedbound	Severe mobility difficulties
13CC.00	Immobile	Severe mobility difficulties
13CD.00	Mobility very poor	Severe mobility difficulties
13CE.00	Mobility poor	Severe mobility difficulties
398A.00	Dependent on helper pushing wheelchair	Severe mobility difficulties
8D9..13	Wheel chair	Severe mobility difficulties

8D92.00	Self propelled wheel chair	Severe mobility difficulties
8D93.00	Pedal powered wheel chair	Severe mobility difficulties
8D94.00	Powered wheel chair	Severe mobility difficulties
8D95.00	Wheel chair unspecified	Severe mobility difficulties
8D9A.00	Attendant powered wheel chair	Severe mobility difficulties
8D9B.00	Wheel chair seating	Severe mobility difficulties
8HHC.00	Referred for wheelchair assessment	Severe mobility difficulties
8N1..00	Modification of wheelchair	Severe mobility difficulties
8T0M.00	Referral to wheelchair service	Severe mobility difficulties
9R43.00	Wheelchair in need of repair	Severe mobility difficulties
9R44.00	Wheelchair in good repair	Severe mobility difficulties
9RA..00	Wheelchair applied for	Severe mobility difficulties
F240.00	Quadriplegia	Severe mobility difficulties
F240.11	Tetraplegia	Severe mobility difficulties
F240100	Spastic tetraplegia	Severe mobility difficulties
F241.00	Paraplegia	Severe mobility difficulties
F241100	Spastic paraplegia	Severe mobility difficulties
F242.00	Diplegia of upper limbs	Severe mobility difficulties
F243.00	Monoplegia of lower limb	Severe mobility difficulties
F244.00	Monoplegia of upper limb	Severe mobility difficulties
N233100	Immobility syndrome	Severe mobility difficulties
R00A.00	[D]Poor mobility	Severe mobility difficulties
R00C.00	[D]Immobility	Severe mobility difficulties
U105.00	[X]Fall involving wheelchair	Severe mobility difficulties
U105000	[X]Fall involving wheelchair, occurrence at home	Severe mobility difficulties
U105100	[X]Fall involvng wheelchair occurrence residential instit'n	Severe mobility difficulties
U105200	[X]Fall invlv w'chair occ school oth instit/pub admin area	Severe mobility difficulties
U105500	[X]Fall involvng wheelchair occurrnce at trade/service area	Severe mobility difficulties
U105700	[X]Fall involving wheelchair, occurrence on farm	Severe mobility difficulties
U105y00	[X]Fall involv wheelchair, occurrnce at other specif place	Severe mobility difficulties
U105z00	[X]Fall involving wheelchair occurrnce at unspecified place	Severe mobility difficulties
Z6R3.00	Wheelchair dancing therapy	Severe mobility difficulties
Z6R8100	Wheelchair sport	Severe mobility difficulties
Z6X1.00	Wheelchair transfer practice	Severe mobility difficulties
Z6Z..00	Wheelchair education	Severe mobility difficulties
Z6Z1.00	Wheelchair use training	Severe mobility difficulties
Z6Z1200	Propelling wheelchair training	Severe mobility difficulties
Z6Z1300	Controlling electric wheelchair training	Severe mobility difficulties
Z9EH400	Provision of wheelchair	Severe mobility difficulties
ZO2..00	Unable to mobilise	Severe mobility difficulties
ZO4..00	Does not mobilise	Severe mobility difficulties
ZO72.00	Unable to mobilise indoors	Severe mobility difficulties
ZO74.00	Does not mobilise indoors	Severe mobility difficulties
ZO75.00	Difficulty mobilising indoors	Severe mobility difficulties
ZO82.00	Unable to mobilise outside	Severe mobility difficulties

ZO84.00	Does not mobilise outside	Severe mobility difficulties
ZO92.00	Unable to mobilise using mobility aids	Severe mobility difficulties
ZO94.00	Does not mobilise using mobility aids	Severe mobility difficulties
ZO96.00	Ability to mobilise using wheelchair	Severe mobility difficulties
ZO96.11	Wheelchair mobility	Severe mobility difficulties
ZO96100	Able to mobilise using wheelchair	Severe mobility difficulties
ZO96200	Unable to mobilise using wheelchair	Severe mobility difficulties
ZO96300	Does mobilise using wheelchair	Severe mobility difficulties
ZO96311	Mobilises using wheelchair	Severe mobility difficulties
ZO96400	Does not mobilise using wheelchair	Severe mobility difficulties
ZO96500	Difficulty mobilising using wheelchair	Severe mobility difficulties
ZOC6200	Unable to get in and out of a chair	Severe mobility difficulties
ZOC6400	Does not get in and out of a chair	Severe mobility difficulties
ZOC8200	Unable to get out of a chair	Severe mobility difficulties
ZOC8400	Does not get out of a chair	Severe mobility difficulties
ZOC9200	Unable to get on and off a bed	Severe mobility difficulties
ZOC9400	Does not get on and off a bed	Severe mobility difficulties
ZOCA200	Unable to get on a bed	Severe mobility difficulties
ZOCB200	Unable to get off a bed	Severe mobility difficulties
ZOCB400	Does not get off a bed	Severe mobility difficulties
ZOD2.00	Unable to move in bed	Severe mobility difficulties
ZOD4.00	Does not move in bed	Severe mobility difficulties
ZOD6200	Unable to roll over in bed	Severe mobility difficulties
ZOD6211	Unable to turn over in bed	Severe mobility difficulties
ZOD7200	Unable to turn onto side in bed	Severe mobility difficulties
ZOD8200	Unable to move up and down bed	Severe mobility difficulties
ZV46200	[V]Dependence on wheelchair	Severe mobility difficulties
ZV4L011	[V] Poor mobility	Severe mobility difficulties
ZV53800	[V]Fitting or adjustment of wheelchair	Severe mobility difficulties
F137.11	Athetoid cerebral palsy	Cerebral palsy
F137.12	Athetosis - congenital	Cerebral palsy
F137000	Athetoid cerebral palsy	Cerebral palsy
F137010	Vogt's disease	Cerebral palsy
F137100	Double athetosis	Cerebral palsy
F137111	Congenital athetosis	Cerebral palsy
F137y00	Other specified symptomatic torsion dystonia	Cerebral palsy
F137z00	Symptomatic torsion dystonia NOS	Cerebral palsy
F23..00	Congenital cerebral palsy	Cerebral palsy
F23..11	Congenital spastic cerebral palsy	Cerebral palsy
F23..12	Infantile cerebral palsy	Cerebral palsy
F23..13	Littles disease	Cerebral palsy
F23..14	Cerebral atonia	Cerebral palsy
F230.00	Congenital diplegia	Cerebral palsy
F230.11	Paraplegia - congenital	Cerebral palsy
F230000	Congenital paraplegia	Cerebral palsy
F230100	Cerebral palsy with spastic diplegia	Cerebral palsy

F230111	Spastic diplegic cerebral palsy	Cerebral palsy
F230z00	Congenital diplegia NOS	Cerebral palsy
F231.00	Congenital hemiplegia	Cerebral palsy
F232.00	Congenital quadriplegia	Cerebral palsy
F232.11	Tetraplegia - congenital	Cerebral palsy
F233.00	Congenital monoplegia	Cerebral palsy
F233.11	Congenital spastic foot	Cerebral palsy
F234.00	Infantile hemiplegia NOS	Cerebral palsy
F23y.00	Other congenital cerebral palsy	Cerebral palsy
F23y000	Ataxic infantile cerebral palsy	Cerebral palsy
F23y100	Flaccid infantile cerebral palsy	Cerebral palsy
F23y200	Spastic cerebral palsy	Cerebral palsy
F23y300	Dyskinetic cerebral palsy	Cerebral palsy
F23y400	Ataxic diplegic cerebral palsy	Cerebral palsy
F23y500	Worster-Drought syndrome	Cerebral palsy
F23y511	Congenital suprabulbar paresis	Cerebral palsy
F23y600	Choreoathetoid cerebral palsy	Cerebral palsy
F23yz00	Other infantile cerebral palsy NOS	Cerebral palsy
F23z.00	Congenital cerebral palsy NOS	Cerebral palsy
F2B..00	Cerebral palsy	Cerebral palsy
F2B0.00	Spastic quadriplegic cerebral palsy	Cerebral palsy
F2B1.00	Spastic hemiplegic cerebral palsy	Cerebral palsy
F2B2.00	Bilateral spastic cerebral palsy	Cerebral palsy
F2By.00	Other cerebral palsy	Cerebral palsy
F2Bz.00	Cerebral palsy NOS	Cerebral palsy
F2Bz.00	Cerebral palsy NOS	Cerebral palsy
Fyu9.00	[X]Cerebral palsy and other paralytic syndromes	Cerebral palsy
Fyu9000	[X]Other infantile cerebral palsy	Cerebral palsy
G669.00	Cerebral palsy, not congenital or infantile, acute	Cerebral palsy
7615.11	Gastrojejunostomy, transposed	PEG feeding
7617.00	Gastrostomy operations	PEG feeding
7617.12	Creation of gastrostomy	PEG feeding
7619.11	Gastrotomy NEC	PEG feeding
7632.00	Jejunostomy	PEG feeding
7607211	Insertion of feeding tube via artificial oesophageal opening	PEG feeding
7617000	Creation of permanent gastrostomy	PEG feeding
7617111	Creation of gastrostomy NEC	PEG feeding
7617200	Reconstruction of gastrostomy	PEG feeding
7617400	Attention to gastrostomy tube	PEG feeding
7617500	Removal of gastrostomy tube	PEG feeding
7617600	Change of gastrostomy tube	PEG feeding
7617700	Maintenance of percutaneous endoscopic gastrostomy tube	PEG feeding
7632000	Creation of jejunostomy	PEG feeding
7632100	Refashioning of jejunostomy	PEG feeding
7632300	Creation of feeding jejunostomy	PEG feeding

7822012	Rodney - Smith hepatojejunostomy+insertion tubal prosthesis	PEG feeding
7617y00	Other specified gastrostomy operation	PEG feeding
7617z00	Gastrostomy operation NOS	PEG feeding
761A200	Open insertion of feeding tube into stomach	PEG feeding
761E400	Permanent percutaneous endoscopic gastrostomy	PEG feeding
761E600	Fibreoptic endoscopic percutaneous insert gastrostomy (PEG)	PEG feeding
761E900	Fibreoptic endoscopic removal of gastrostomy tube	PEG feeding
761EA00	Fibreoptic endoscopic percutaneous insertion of gastrostomy	PEG feeding
7632y00	Other specified jejunostomy	PEG feeding
7632z00	Jejunostomy NOS	PEG feeding
8CJ2.00	Percutaneous endoscopic gastrostomy feeding	PEG feeding
8CJ3.00	Jejunostomy feeding	PEG feeding
8CJ4.00	Button gastrostomy feeding	PEG feeding
8D69.00	Gastrostomy aid training	PEG feeding
8D6A.00	Gastrostomy bag fitting	PEG feeding
8D6B.00	Gastrostomy bag adjustment	PEG feeding
8D6C.00	Gastrostomy bag changed	PEG feeding
J524100	Complication of gastrostomy	PEG feeding
TB03300	Formation of gastrostomy with complication, without blame	PEG feeding
ZC32.54	PEG - Percutaneous endoscopic gastrostomy feeding	PEG feeding
ZC65.00	Enteral feeding	PEG feeding
ZC65100	Enteral tube feeding	PEG feeding
ZC65111	Tube feeding	PEG feeding
ZC65112	TF - Tube feeding	PEG feeding
ZC65200	Gastrostomy feeding	PEG feeding
ZC65300	Percutaneous endoscopic gastrostomy feeding	PEG feeding
ZC65311	PEG - Percutaneous endoscopic gastrostomy feeding	PEG feeding
ZC65400	Button gastrostomy feeding	PEG feeding
ZC65500	Jejunostomy feeding	PEG feeding
ZV44100	[V]Has gastrostomy	PEG feeding
ZV55100	[V]Attention to gastrostomy	PEG feeding
**ICD-10 codes from Hospital Episode statistics to identify severe health needs**		
**ICD-10**	**ICD-10 description**	**Interpretation**
G40	Epilepsy	Epilepsy
G41	Status epilepticus	Epilepsy
G06.8	Epileptic psychosis NOS	Epilepsy
F80.3	Acquired aphasia with epilepsy [Landau-Kleffner]	Epilepsy
N39.3	Stress incontinence	Incontinence
N39.4	Other specified urinary incontinence	Incontinence
R15	Foecal incontinence	Incontinence
R32	Unspecified urinary incontinence	Incontinence
F98.0	Nonorganic enuresis	Incontinence
F98.1	Nonorganic encopresis	Incontinence
H54.0	Blindness, binocular	Severe visual impairment

H54.1	Severe visual impairment, binocular	Severe visual impairment
H54.4	Blindness, monocular	Severe visual impairment
H54.5	Severe visual impairment, monocular	Severe visual impairment
H90	Conductive and sensorinueral hearing loss	Severe hearing impairment
H93.0	Transient ischaemic deafness	Severe hearing impairment
H91.0	Ototoxic hearing loss	Severe hearing impairment
H91.2	Sudden idiopathic hearing loss	Severe hearing impairment
H91.3	Deaf mutism, not elsewhere classified	Severe hearing impairment
G82	Paraplegia and tetraplegia	Severe mobility difficulties
G83.1	Monoplegia of lower limb	Severe mobility difficulties
G83.5	Locked-in syndrome	Severe mobility difficulties
Z46.8	Fitting and adjustment of other specified device - wheelchair	Severe mobility difficulties
W05	Fall involving wheelchair	Severe mobility difficulties
Z99.3	Dependence of wheelchair	Severe mobility difficulties
Z74.0	Need for assistance due to reduced mobility	Severe mobility difficulties
R26.3	Immobility	Severe mobility difficulties
G80	Cerebral palsy	Cerebral palsy
Z43.1	Attention to gastrostomy	PEG feeding
Z93.1	Gastrostomy status	PEG feeding

Figure S1: Data flow diagram of individuals included in the study population from original extracted data[a]

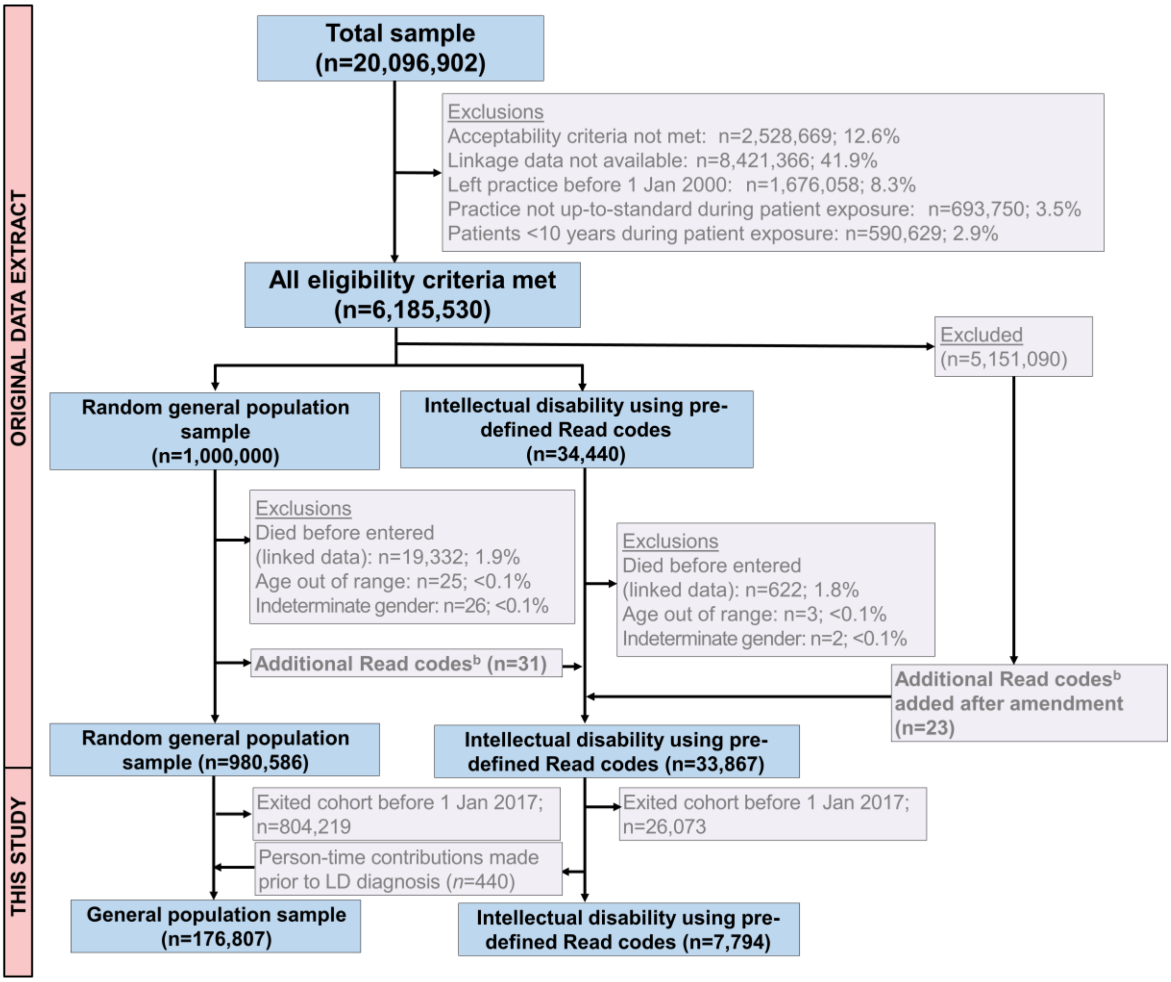

[a] For the original extraction, an unstratified random sample was selected. Age (median 34.73yrs vs 34.74yrs), gender (48.39% vs 48.36%), proportion of deaths (estimated from CPRD data prior to deaths linkage data availability; 7.12% vs 7.13%), and length of time in the cohort (also estimated prior to linkage data availability; 5.10 vs 5.09yrs) for the selected vs entire population were compared to ensure that no obvious differences were evident.

[b] Additional Read codes for Angelman and Cockayne syndrome added

Table S3: Characteristics of individuals with specific health needs under investigation

Characteristics		Intellectual disability Number/ Median	Percent/ Range	No intellectual disability[a] Number/ Median	Percent/ Range
**Epilepsy present (baseline/follow-up)**					
Total		1,511	100.0	2,205	100.0
Age (years)		39.0	10–86	48.8	10–103
Gender:	Male	871	57.6	1131	51.3
	Female	640	42.4	1074	48.7
Ethnicity:	White	1,325	87.7	1,909	86.6
	South Asian	41	2.7	52	2.4
	Black African/Caribbean	33	2.2	51	2.3
	Other	44	2.9	72	3.3
	Not known	68	4.5	121	5.5

Length in cohort (years):	1.6 >0.0–2.7	1.5 >0.0–2.7
Most common syndromes[b]:<br>No cause identified<br>Down syndrome<br>Tuberous sclerosis<br>Rett syndrome<br>Angelman syndrome	 1,320 87.4<br>82 5.4<br>42 2.8<br>15 1.0<br>13 0.9	
Other health needs (baseline/follow-up):<br>Incontinence<br>Severe visual loss<br>Severe hearing impairment<br>Severe mobility difficulties<br>Cerebral palsy<br>PEG feeding	 422 27.9<br>382 25.3<br>121 8.0<br>499 33.0<br>359 23.8<br>98 6.5	 256 11.6<br>68 3.1<br>111 5.0<br>153 6.9<br>70 3.2<br>34 1.5
**Incontinence present (baseline/follow-up)**		
Total	1,253 100.0	7,826 100.0
Age (years)	45.0 10–96	57.0 10–106
Gender: Male<br>Female	587 46.9<br>666 53.2	2,091 26.7<br>5,735 73.3
Ethnicity: White<br>South Asian<br>Black African/Caribbean<br>Other<br>Not known	1,070 85.4<br>34 2.71<br>36 2.87<br>39 3.11<br>74 5.91	6,568 83.9<br>269 3.4<br>218 2.8<br>289 3.7<br>482 6.2
Length in cohort (years):	1.4 >0.0–2.7	1.6 >0.0–2.7
Most common syndromes[b]:<br>No cause identified<br>Down syndrome<br>Fragile X syndrome<br>Tuberous sclerosis<br>Prader-Willi syndrome	 1,046 83.6<br>135 10.8<br>9 0.7<br>8 0.6<br>7 0.6	
Other health needs (baseline/follow-up):<br>Epilepsy<br>Severe visual loss<br>Severe hearing impairment<br>Severe mobility difficulties<br>Cerebral palsy<br>PEG feeding	 422 33.7<br>322 25.7<br>111 8.9<br>413 33.0<br>215 17.2<br>65 5.2	 256 3.3<br>186 2.4<br>594 7.6<br>581 7.4<br>40 0.5<br>44 0.6
**Severe visual loss present (baseline/follow-up)**		
Total	1,242 100.0	1,279 100.0
Age (years)	46.0 10–88	73.2 (10–107)
Gender: Male<br>Female	682 54.9<br>560 45.1	624 48.8<br>655 51.2
Ethnicity: White<br>South Asian<br>Black African/Caribbean<br>Other<br>Not known	1,011 81.4<br>34 2.7<br>38 3.1<br>42 3.4<br>117 9.4	1,083 84.7<br>39 3.1<br>46 3.6<br>48 3.8<br>63 4.9
Length in cohort (years):	1.6 >0.0–2.7	1.5 >0.0–2.7
Most common syndromes[b]:<br>No cause identified<br>Down syndrome	 1,039 83.7<br>165 13.3	
Other health needs (baseline/follow-up):<br>Epilepsy<br>Incontinence	 382 30.8<br>322 25.9	 68 5.3<br>186 14.5

Severe hearing impairment	192	15.5	176	13.8
Severe mobility difficulties	370	29.8	127	9.9
Cerebral palsy	205	16.5	12	0.9
PEG feeding	42	3.4	11	0.9
**Severe hearing loss present (baseline/follow-up)**				
Total	618	100.0	5,845	100.0
Age (years)	43.0	10–91	69.0	10–107
Gender: Male	343	55.5	2,968	50.8
Female	275	44.5	2,877	49.2
Ethnicity: White	521	84.3	4,935	84.4
South Asian	18	2.9	115	2.0
Black African/Caribbean	8	1.3	56	1.0
Other	21	3.4	136	2.3
Not known	50	8.1	603	10.3
Length in cohort (years):	1.5	>0.0–2.7	1.8	>0.0–2.7
Most common syndromes[b]:				
No cause identified	432	69.9		
Down syndrome	128	20.7		
Other health needs (baseline/follow-up):				
Epilepsy	121	19.6	110	1.9
Incontinence	111	18.0	594	10.2
Severe visual loss	413	33.0	176	3.0
Severe mobility difficulties	113	18.3	208	3.6
Cerebral palsy	53	8.6	11	0.2
PEG feeding	14	2.3	26	0.4
**Severe mobility difficulties present (baseline/follow-up)**				
Total	992	100.0	1,850	100.0
Age (years)	47.0	10–96	76.6	10–107
Gender: Male	521	52.5	745	40.3
Female	471	47.5	1,105	59.7
Ethnicity: White	868	87.5	1,681	90.9
South Asian	32	3.2	46	2.5
Black African/Caribbean	25	2.5	49	2.7
Other	25	2.5	47	2.5
Not known	42	4.2	27	1.5
Length in cohort (years):	1.4	>0.0–2.7	0.9	>0.0–2.7
Most common syndromes[b]:				
No cause identified	862	86.9		
Down syndrome	79	8.0		
Rett syndrome	12	1.2		
Other health needs (baseline/follow-up):				
Epilepsy	499	50.3	153	8.3
Incontinence	413	41.6	581	31.4
Severe visual loss	370	37.3	127	6.9
Severe hearing impairment	113	11.4	208	11.2
Cerebral palsy	378	38.1	67	3.6
PEG feeding	107	10.8	66	3.6
**Cerebral palsy present (baseline/follow-up)**				
Total	678	100.0	267	100.0
Age (years)	33.0	10–78	28.0	10–89
Gender: Male	396	58.4	161	60.3
Female	282	41.6	106	39.7
Ethnicity: White	570	84.1	222	83.2
Other	74	10.9	21	7.9

Not known	34	5.0	24	9.0
Length in cohort (years):	1.7	>0.0–2.7	1.6	>0.0–2.7
Most common syndromes[b]:				
No cause identified	655	96.6		
Other health needs (baseline/follow-up):				
Epilepsy	359	53.0	70	26.2
Incontinence	215	31.7	40	15.0
Severe visual loss	205	30.2	12	4.5
Severe hearing impairment	53	7.8	12	4.5
Severe mobility difficulties	378	55.8	67	25.1
PEG feeding	84	12.4	17	6.4
**PEG feeding tube present (baseline/follow-up)**				
Total	152	100.0	324	100.0
Age (years)	23.0	10–74	62.5	10–97
Gender: Male	85	55.9	149	63.7
Female	67	44.1	85	36.3
Ethnicity: White	136	89.5	211	90.2
Other/not known	16	10.5	23	9.8
Length in cohort (years):	1.4	>0.0–2.7	1.3	>0.0–2.7
Most common syndromes[b]:				
No cause identified	126	82.9		
Down syndrome	10	6.6		
Other health needs (baseline/follow-up):				
Epilepsy	98	64.5	34	14.5
Incontinence	65	42.8	44	18.8
Severe visual loss	42	27.6	11	4.7
Severe hearing impairment	14	9.2	27	11.5
Severe mobility difficulties	107	70.4	66	28.2
Cerebral palsy	84	55.3	17	7.3
**No health needs (at baseline or follow-up)**				
Total	4,174	100.0	159,716	100.0
Age (years)	28.0	10–101	42.0	10–108
Gender: Male	1,701	59.3	79,760	49.9
Female	2,473	40.8	79,956	50.1
Ethnicity: White	2,974	71.3	104,982	65.7
South Asian	102	2.4	7,184	4.5
Black African/Caribbean	122	2.9	5,868	3.7
Other	163	3.9	8,362	5.2
Not known	813	19.5	33,320	20.9
Length in cohort (years):	1.2	>0.0–2.7	1.8	>0.0–2.7
Most common syndromes[b]:				
No cause identified	2,726	65.3		
Down syndrome	460	11.0		
Fragile X syndrome	125	3.0		
Edward syndrome	25	0.6		
Tuberous sclerosis	16	0.4		

PEG: percutaneous endoscopic gastrostomy

[a] Includes individuals that switched intellectual disability status during observation period (epilepsy: *n*=68; incontinence: *n*=40; severe visual loss: *n*=12; severe hearing impairment: *n*=10; severe mobility difficulties: *n*=18; cerebral palsy: *n*=37; PEG: *n*=7; no health needs: *n*=280)

[b] Figures only reported where >5 individuals
